# Assessment of Quality of Life in the Early Postoperative Period in Patients with Lung Cancer

**DOI:** 10.3390/ijerph22121810

**Published:** 2025-11-30

**Authors:** Stana Pačarić, Tajana Turk, Ivan Erić, Marko Babić, Suzana Luketić, Nika Srb, Andrea Milostić Srb, Anamarija Petek Erić, Mario Duvnjak

**Affiliations:** 1 Faculty of Dental Medicine and Health Osijek, Nursing Institute “Professor Radivoje Radić”, Josip Juraj Strossmayer University of Osijek, 31000 Osijek, Croatia; stana.pacaric@gmail.com (S.P.); suzana.luketic@kbco.hr (S.L.); milostic.andrea@gmail.com (A.M.S.); 2 University Hospital Centre Osijek, 31000 Osijek, Croatia; ivan.eric@kbco.hr (I.E.); anamarija.petek-eric@kbco.hr (A.P.E.); mario.duvnjak@kbco.hr (M.D.); 3 Faculty of Medicine, Josip Juraj Strossmayer University in Osijek, 31000 Osijek, Croatia

**Keywords:** cancer, lung cancer, NSCLC, SCLC, surgery, quality of life

## Abstract

Measuring quality of life (QoL) in patients with early-stage lung cancer is an important aspect of treatment success. This study assessed QoL in the early postoperative period in patients with lung cancer, with regard to the type of cancer. This single-center study was conducted on 64 patients who underwent surgery for non-small cell (NSCLC) and small cell (SCLC) lung cancer. Quality of life questionnaires (QLQs) of the European Organization for Research and Treatment of Cancer (EORTC) were used. The EORTC QLQ-C30 questionnaire assesses the quality of life of cancer patients, and the EORTC QLQ-LC13 questionnaire is a lung cancer module. In the group of patients with NSCLC, the general health status (*p* < 0.001), physical functioning (*p* = 0.004), emotional functioning (*p* = 0.005) and total functional scale (*p* = 0.01) were significantly better assessed, fatigue was less pronounced (*p* = 0.005), nausea/vomiting (*p* = 0.04), pain (*p* = 0.004), breathing difficulties were less pronounced (*p* = 0.03), loss of appetite was less pronounced (*p* = 0.005), and the symptom scale was significantly less pronounced (*p* = 0.002) compared to patients with SCLC. In the QLQ-LC13 symptom scale, SCLC patients had more cough (*p* = 0.02), dyspnea (*p* = 0.03), dysphagia (*p* = 0.005), peripheral neuropathy (*p* = 0.04), chest pain (*p* < 0.001), arm or shoulder pain (*p* < 0.001), and pain in other parts of the body (*p* = 0.005) compared to NSCLC patients. Patients with NSCLC evaluated the functioning scales better and had less pronounced symptoms on the symptom scale, while patients with SCLC evaluated the treatment symptoms worse on the symptom scale, especially the symptom of pain, which had an impact on the quality of life of the patients. The results of this study could contribute to raising public awareness about the quality of life of lung cancer patients.

## 1. Introduction

According to data from the World Health Organization, lung cancer remains one of the most prevalent malignant diseases and the leading cause of cancer-related deaths worldwide. In Croatia, approximately 3000 new cases are diagnosed each year, the majority of which occur among smokers. In Croatia, lung cancer ranks as the third most frequently diagnosed cancer among men, accounting for 16%, which is slightly above the European Union (EU) average, after prostate and colorectal cancer. Among women, lung cancer also ranks third, at 9%, which is close to the EU average, after breast and colorectal cancer [1]. Approximately 90% of individuals diagnosed with lung cancer are current or former smokers and most of the diagnosed patients die. Data indicate that the five-year survival rate for lung cancer in the United States is approximately 16%, while in Croatia the survival rate is about 10% [2]. Due to the aggressive nature of this disease, the Croatian Oncological Society of the Croatian Medical Association has developed national guidelines for the diagnosis, treatment, and follow-up of patients with lung cancer.

The initial treatment plan is made by a multidisciplinary team consisting of a radiologist, pulmonologist, pathologist, oncologist and thoracic surgeon [3]. To help reduce lung cancer mortality, Croatia has introduced a National Lung Cancer Screening Program, which began in 2020, to enable the early diagnosis of cancer patients, thus increasing the possibility of treatment, improving survival and reducing mortality [4]. In implementing the guidelines, the use of low-dose computed tomography (LDCT) is strongly recommended, as it enables the detection of lung cancer at an early stage without significant radiation exposure. Croatia is one of the countries that has committed to the widespread implementation of targeted LDCT screening, along with Poland, Italy, Romania, Belgium, Germany, France, and Spain [3,4].

Clinically, primary lung cancer is classified into two main types: non-small cell lung cancer (NSCLC) and small cell lung cancer (SCLC). NSCLC represents approximately 85% of all lung cancer cases. The primary risk factor for NSCLC is smoking, although environmental and genetic factors also contribute to its development. Treatment options vary depending on the stage of the disease and may include surgery, radiation therapy, chemotherapy, and targeted therapy. With advances in genetics and biomarker testing, specific mutations have been identified, enabling personalized, targeted treatment approaches for individual patients.

SCLC accounts for approximately 15% of all lung cancer cases and is also strongly linked to smoking. Symptoms usually develop rapidly, starting 8 to 12 weeks before the onset of lung cancer. The signs and symptoms vary depending on the location and size of the primary tumor. They may include cough, wheezing, and hemoptysis. Treatment largely depends on the stage of the disease. Patients with limited-stage SCLC are candidates for radiation therapy and chemotherapy with the intent to cure. Since SCLC has a very poor prognosis, currently the emphasis is on screening and prevention. There is considerable evidence that smoking cessation can reduce the incidence of SCLC [5,6,7].

Quality of life (QoL) encompasses overall well-being as well as physical, psychological, and social dimensions. QoL assessments should be sensitive enough to detect clinically meaningful changes resulting from treatment. In patients with lung cancer, QoL is influenced by multiple factors, including individual patient characteristics, disease stage, and treatment-related factors. Despite advances, there has been limited improvement in survival rates in recent years, making the impact of treatment on quality of life increasingly important [8,9]. Research on the quality of life (QoL) of lung cancer patients is limited, largely because this patient group typically has a poor long-term survival rate, as the disease is often diagnosed at an advanced stage and considered incurable. Lung cancer patients experience a range of distressing symptoms that can significantly impair their quality of life. When it comes to symptoms, studies have shown that patients report pain, fatigue, cough, dyspnea, in addition to physical symptoms, patients with lung cancer experience psychological problems such as sadness, anxiety, which negatively affect emotions related to QoL. Patients with lung cancer reported poorer self-rated health compared with those with breast or colon cancer [10]. Research indicates that patients who struggle to accept a lung cancer diagnosis prior to surgery tend to experience a higher symptom burden and poorer overall health status [11,12]. Although many studies have assessed quality of life in lung cancer patients using the EORTC QLQ-C30 and QLQ-LC13, few have directly examined correlations between postoperative symptom burden and QoL. Reporting such relationships could clarify how specific symptoms affect different QoL domains and guide targeted interventions in postoperative care. This is the first study in Croatia to evaluate the quality of life of lung cancer patients during the early postoperative period.

The aim of this study is to examine the QoL in the early postoperative period in patients with lung cancer. To examine the QoL of operated patients with lung cancer with regard to the type of cancer, the presence of pneumothorax and smoking status, and to examine the burden of symptoms in relation to the type of cancer. The aim was also to examine the relationship between QoL and the presence of symptoms.

## 2. Materials and Methods

### 2.1. Study Design and Samples

A cross-sectional study was conducted between October 2022 and December 2024 among patients who underwent lung cancer surgery, assessed 1–3 months postoperatively at the Outpatient Clinic of the Department of Thoracic Surgery, Osijek Clinical Hospital Center. The study included patients diagnosed with non-small cell lung cancer (NSCLC) and small cell lung cancer (SCLC). Ethical approval was obtained from the Ethics Committee of the Osijek Clinical Hospital Center (R1-499-2/2022). All participants were informed about the study’s purpose and assured of data anonymity, and participation was entirely voluntary.

Inclusion criteria for the study: patients operated on for lung cancer (patohistological finding of lung cancer), patients who received neoadjuvant therapy before surgery (to reduce the tumor), patients who started oncological therapy after surgery, patients older than 18 years, patients who could speak and read Croatian.

The exclusion criteria were age under 18 years, life expectancy of less than one year, presence of cognitive and/or mental disorders, illiteracy, and inability to communicate in Croatian. Of the 98 patients who underwent lung cancer surgery during the study period, 75 met the inclusion criteria. During the study, 5 patients withdrew their consent, and 6 provided incomplete questionnaires, resulting in a final sample of 64 participants.

### 2.2. Demographic Data

The sociodemographic questionnaire consists of sociodemographic characteristics (gender, age, place of residence, level of education, marital status, employment), family history, smoking status and information on the type and stage of cancer, treatment history, chemotherapy, radiotherapy or chemotherapy and radiotherapy. The data were collected from the patient’s medical records.

Patients who participated in the study with NSCLC lung cancer received neoadjuvant therapy to reduce the tumor and were operated on and aw well, and then depending on the values of the markers: EGFR, ALK, PD-L1, started adjuvant therapy.

Patients in the early-stage SCLC study with localized, small, node-free tumors underwent surgery and adjuvant therapy.

Lung resections were generally performed using a muscle-sparing thoracotomy approach. Postoperative management included chest physiotherapy, early mobilization, antibiotic and antithrombotic prophylaxis, and postoperative pain control with continuous intravenous infusion of ketorolac and tramadol, aimed at maintaining a visual analog scale (VAS) score below 3–4 during the first 72 h (VAS range 0–10, assessed twice daily). No structured physiotherapy or psychological support programs were provided prior to surgery.

### 2.3. EORTC QLQ-LC13 and EORTC QLQ-C30 Quality of Life Questionnaire

The study was conducted using the European Organization for Research and Treatment of Cancer (EORTC) QoL questionnaire (QLQ-C30) [13] and the EORTC QLQ-LC13 [14] developed model for lung cancer, both questionnaires were used to assess QoL. Permission to use the validated Croatian versions of both questionnaires was obtained from the EORTC Quality of Life Group, Brussels, Belgium [15].

#### 2.3.1. EORTC QLQ-C30 Quality of Life Questionnaire

The European Organization for Research and Treatment of Cancer Quality of Life Questionnaire (EORTC QLQ-C30) [13] is a cancer-specific, multidimensional instrument designed to assess health-related quality of life in cancer patients. It includes five functional domains (physical, role, emotional, cognitive, and social functioning), three symptom domains (fatigue, nausea/vomiting, and pain), six single items (dyspnea, insomnia, appetite loss, constipation, diarrhea, and financial difficulties), and one global health status/QoL scale. Scores for each domain are linearly transformed to a 0–100 scale according to the EORTC scoring manual. Higher scores on the functional and global health/QoL scales indicate better functioning and quality of life, whereas higher scores on the symptom scales and single items indicate a greater symptom burden or poorer health status.

#### 2.3.2. EORTC QLQ-LC13 Lung Cancer Module

The EORTC QLQ-LC13 [14] is a lung cancer–specific supplementary module developed to complement the core QLQ-C30 questionnaire. It includes 13 items addressing symptoms and treatment-related side effects that may not be adequately captured by the core instrument. The module comprises one multi-item dyspnea scale and several single-item measures assessing cough, hemoptysis, dysphagia, pain in the chest, arm, or other areas, oral pain, alopecia, and peripheral neuropathy. Scores for all LC13 items are also transformed linearly to a 0–100 scale. Higher scores represent a higher level of symptoms or treatment-related problems and, consequently, a poorer quality of life.

### 2.4. Research Questions

The aim of this study was to examine the quality of life (QoL) in the early postoperative period in patients with lung cancer using the EORTC QLQ-C30 and EORTC QLQ-LC13 questionnaires. Specifically, the study investigated whether QoL differed according to the type of lung cancer, the presence of pneumothorax, and smoking status, and whether disease-specific symptoms varied with cancer type or neoadjuvant therapy. Additionally, the relationship between the burden of symptoms and overall QoL was explored. In this context, the independent variables included type of cancer, presence of pneumothorax, smoking status, and neoadjuvant therapy, while the dependent variables were the scores on the QLQ-C30 and QLQ-LC13 scales, reflecting global QoL, functional status, and symptom burden.

### 2.5. Statistical Methods

Categorical variables were presented as absolute and relative frequencies. The normality of the distribution of continuous variables was assessed using the Shapiro–Wilk test. Continuous variables were summarized as medians with interquartile range (IQR) limits. Differences between two independent groups were analyzed using the Mann–Whitney U test, while comparisons among three or more independent groups were performed using the Kruskal–Wallis test. These nonparametric tests were chosen because they do not assume a normal distribution and are appropriate for comparing independent samples when data are not normally distributed. Preliminary analyses of normality (Shapiro–Wilk test) and visual inspection of histograms and Q–Q plots confirmed that the assumptions of these tests were met. The assessment of the connection is given by Spearman’s correlation coefficient [16]. All *p* values are two-sided. The significance level was set at alpha = 0.05. The statistical package MedCalc^®^ Statistical Software version 23.3.4 (MedCalc Software Ltd., Ostend, Belgium; https://www.medcalc.org; 2025, accessed on 5 May 2025) was used for statistical analysis. The research report was prepared in accordance with the guidelines for reporting studies in biomedical and health sciences [17].

## 3. Results

The study was conducted on 64 patients, of whom 54 (84%) were male and 31 (49%) patients were aged 46–65 years. Regarding their place of residence, 41 (64%) of them lived in the city, and according to their level of education, 43 (67%) had a secondary education. 43 (68%) were married, and 28 (44%) were employed. Pneumothorax was recorded in 12 (19%) of patients. Regarding family history, 18 (28%) of the patients had a family history of lung cancer. 25 (39%) of them were current smokers, and 24 (38%) were former smokers. Neoadjuvant therapy was received by 29 (45%) patients, of whom 25/29 (86%) received chemotherapy. NSCLC was present in 54 (84%) of the patients and SCLC was present in 10 (16%) (Table 1).

In the group of patients with NSCLC, compared to patients with SCLC, the general health status (Mann–Whitney U test, *p* < 0.001), physical functioning (Mann–Whitney U test, *p* = 0.004), emotional functioning (Mann–Whitney U test, *p* = 0.005) and the total functionality scale (Mann–Whitney U test, *p* = 0.01) were significantly better assessed, fatigue was less pronounced (Mann–Whitney U test, *p* = 0.005), nausea/vomiting (Mann–Whitney U test, *p* = 0.04), pain (Mann–Whitney U test, *p* = 0.004), breathing difficulties were less severe (Mann–Whitney U test, *p* = 0.03), loss of appetite was less severe as well (Mann–Whitney U test, *p* = 0.005), and the symptom scale was significantly less pronounced (Mann–Whitney U test, *p* = 0.002) (Table 2).

Patients with pneumothorax, compared to other patients, have a significantly worse general health status (Mann–Whitney U test, *p* = 0.04), work functioning (Mann–Whitney U test, *p* = 0.03), and significantly more frequent constipation (Mann–Whitney U test, *p* = 0.02) (Table 3).

Non-smokers have significantly better general health status compared to active or former smokers (Kruskal–Wallis test, *p* = 0.04) and work functioning is marginally significantly higher in the non-smoker group compared with the other two groups (Kruskal–Wallis test, *p* = 0.07). While in functioning and symptoms scale there are no significant differences compared to a smoking status (Table 4).

In the QLQ-LC13 scale, SCLC patients, compared to the NSCLC group, had more pronounced cough (Mann–Whitney U test, *p* = 0.02), dyspnea (Mann–Whitney U test, *p* = 0.03), dysphagia (Mann–Whitney U test, *p* = 0.005), peripheral neuropathy (Mann–Whitney U test, *p* = 0.04), chest pain (Mann–Whitney U test, *p* < 0.001), arms or shoulder pain (Mann–Whitney U test, *p* < 0.001), and pain in other parts of the body (Mann–Whitney U test, *p* = 0.005) (Table 5).

The pain reduction score was significantly higher in patients who did not have preoperative oncological therapy, compared to other patients, if they took painkillers (Mann–Whitney U test, *p* = 0.04) (Table 6).

Spearman’s correlation coefficient was used to assess the correlation between the QLQ-LC13 and QLQL-C30 scales. The negative correlation between cough and work functioning is somewhat stronger (Rho = −0.520) as can be seen in the table. If hemoptysis is more pronounced, the general health status is assessed worse (Rho = −0.378). More pronounced dyspnea is associated with the worse general health status assessment (Rho = −0.443), emotional functioning (Rho = −0.485) and more pronounced pain (Rho = 0.479), while the other significant correlations are somewhat weaker. If oral pain is more pronounced, diarrhea (Rho = 0.357) and nausea/vomiting (Rho = 0.321) are more pronounced. The strongest connection between dysphagia is with diarrhea (Rho = 0.506), and peripheral neuropathy with pain (Rho = 0.426). Alopecia is significantly and slightly better positively correlated with nausea/vomiting (Rho = 0.406) and with the total symptom scale (Rho = 0.413). The more severe the chest pain, the poorer the emotional functioning (Rho = −0.561), and the pain the more pronounced (Rho = 0.568). If patients have more pronounced pain in the arms and shoulders, their general health status is poorer (Rho = −0.471), emotional functioning is lower (Rho = −0.488), cognitive functioning is lower (Rho = −0.525), and pain (Rho = 0.429) and breathing difficulties (Rho = 0.436) are greater. If pain in other parts of the body is more pronounced, the functionality scale is lower (Rho = −0.530) and the symptom scale is more pronounced (Rho = 0.590) (Table 7).

## 4. Discussion

The study included the measurement of QoL in patients with lung cancer and the results showed that the general health status on the QLQ-C30 scale was significantly better rated by patients with NSCLC compared to those with SCLS. These results are in line with the treatment strategies for NSCLC which depend on the stage of the disease and range from surgery to palliative chemotherapy, while in the treatment of SCLC the treatment is generally aggressive and primarily based on oncological therapy [18,19]. In patients with SCLC, the decline in global QoL is influenced by several factors related to the patient, the disease and the characteristics of the treatment. Such factors are correlated with the type and stage of the disease that influence the different treatment strategies that are combined with the combination chemoradiotherapy used to treat limited SCLC, which causes side effects such as cough, hemoptysis, chest pain, anorexia and dysphagia, which may affect the assessment of global QoL in patients with SCLC [20].

Findings from studies conducted worldwide have explored the relationship between quality of life (QoL) and survival. However, it remains uncertain whether global QoL is a stronger predictor of survival than physical QoL. Moreover, when QoL is considered not only as a potential predictor of survival but also as an indicator of patient vulnerability, interpreting global QoL scores can be challenging due to their broad and multifaceted nature [21,22].

The Functionality Scale in NSCLC patients has better assessment results while the Symptom Scale has significantly more pronounced symptoms in the SCLC group in this study. Physical, emotional, and social functioning were rated significantly higher by patients with NSCLC. Assessing patients’ ability to perform daily activities, maintain independence, and cope with fatigue or dyspnea is essential for identifying difficulties and implementing targeted interventions. In contrast, patients with early-stage SCLC often undergo multiple treatment modalities, including surgery, radiotherapy, and chemotherapy, which may contribute to a higher symptom burden [23,24,25]. Therefore, their QoL largely depends on the support network for help or supervision during physical activities, such as walking where fatigue occurs, which has an impact on other functioning scales and QoL [26]. Substantial care and assistance from family members, particularly caregivers such as spouses or children who provide help with daily activities contributes to reduced suffering and better quality of life for individuals with lung cancer [27,28].

The patients that experienced pneumothorax complication had worse self-rated general health status, cognitive functioning, and significantly more frequent constipation. In this study, 19% of patients had pneumothorax. The fear and pain linked to pneumothorax increase patients’ overall stress levels and stem from factors such as the initial onset of pain, the emergency room experience, diagnostic procedures, thoracic drain insertion, and any required corrective surgery.

Constipation is a frequent side effect in lung cancer patients undergoing chemotherapy and is also commonly associated with opioid use. Once constipation occurs, it significantly affects the physical and mental health of patients and consequently affects the QoL, therefore it is important to advise on dietary changes and prescribe laxatives as needed to prevent adverse health effects [29,30].

Smoking is a well-established risk factor for numerous diseases, including lung cancer, COPD, coronary heart disease, stroke, and peripheral vascular disease [30]. Both current and former smokers face a significantly higher risk of developing lung cancer compared with nonsmokers, although this risk declines over time after smoking cessation. Tobacco use not only contributes to the onset of lung cancer but also adversely influences its treatment outcomes [31]. Research has highlighted the influence of psychosocial factors on continued smoking, with depressive symptoms, emotional distress, low perceived risk, and low self-efficacy to quit all playing important roles. Socioeconomic factors such as family smoking patterns, low income, and limited education also contribute to the likelihood of ongoing smoking [32,33]. In this study, 39% of the patients currently smoke and 38% of them had smoked or quit smoking at some time before the study, with nonsmokers assessing their health status better than smokers and former smokers. Smoking is one of the most dominant risk factors in the prognosis of SCLC and NSCL [29]. Smoking cessation remains a critical component of postoperative care for patients with lung cancer, as continued smoking is associated with poorer treatment outcomes, slower recovery, and reduced quality of life. Integrating structured cessation support into routine oncological care can significantly improve patient outcomes. Practical measures include brief and repeated counseling, pharmacological support and access to psychological assistance for managing stress and dependence. Embedding these interventions within a multidisciplinary framework linking oncologists, pulmonologists, nurses, and mental health professionals can enhance adherence and long-term abstinence. Digital tools, telemedicine follow-ups, and involvement of family members may further strengthen cessation efforts and support sustained behavioral change [32,33].

The QLQ-LC13 symptom scale has shown that patients with lung cancer undergoing surgery experience side effects and symptoms that affect their QoL. Studies have demonstrated that symptom burden can persist long after surgery. Findings show that acute postoperative pain is very common and typically intensifies during the first 1–3 months compared with the immediate postoperative period, after which it gradually decreases for some patients, though the pattern varies [34]. Chronic pain usually lasts over two-three months, it is also known as post-thoracotomy pain syndrome, and is often shoulder or neuropathic in nature, affecting 30% of patients after surgery and in some reports up to 40% of patients after minimally invasive surgery [34]. Symptoms of pain after surgery are associated with anxiety, if pain is not controlled, it can affect mental health by developing symptoms related to post-traumatic stress disorder in up to 50% of patients operated on for lung cancer, and pain is the symptom that worries them the most because it can occur in multiple locations at the same time [35,36,37]. Inadequately managed pain can impair mobility, limit respiratory function, increase the risk of postoperative complications, and negatively influence emotional well-being. These findings highlight the need for systematic pain assessment using validated tools, early identification of patients at risk for persistent pain, and the implementation of multimodal analgesia strategies. Interventions may include a combination of pharmacological options (opioids, nonsteroidal anti-inflammatory drugs (NSAIDs), regional anesthesia techniques) and non-pharmacological approaches (physiotherapy, breathing exercises, psychological support). A multidisciplinary care model integrating surgeons, anesthesiologists, pulmonologists, oncology nurses, and pain specialists is essential to ensure individualized pain management plans and improve postoperative recovery trajectories [36].

Respiratory symptoms such as dyspnea and cough are very common and often persistent in patients with lung cancer. Chronic cough frequently occurs after lung resection, with studies reporting its presence in approximately 30–40% of postoperative patients. This ongoing cough can lead to complications including fatigue, insomnia, hoarseness, urinary incontinence, rib fractures, and even spontaneous pneumothorax. Furthermore, postoperative cough, particularly chronic cough, can intensify incisional pain after lung surgery and may cause patients to question the effectiveness of their treatment. In some cases, it can even contribute to depressive symptoms, significantly diminishing postoperative quality of life [38,39]. Symptoms in patients operated on for lung cancer also occur in patients who have received or are undergoing oncological therapy.

Because postoperative symptoms and complications are particularly common during the first three months after lung surgery, patients’ quality of life can be markedly reduced by factors such as pain intensity, cough, shortness of breath, fatigue, sleep disturbances, and other physiological symptoms, these findings align with the results of this study [40].

The results of this research showed that pain has a significant impact on the QoL of lung cancer patients. According to the results of this study, pain in other parts of the body had a medium-strong negative relationship with the functionality scales, meaning that patients with pronounced pain have a weaker ability to perform daily activities. In addition to pain, the QoL of these patients was significantly affected by dyspnea (one of the most common symptoms in patients with lung cancer), along with fatigue, which is one of the predictors of reduced QoL. Fatigue is negatively related to emotional and cognitive functioning, which means that patients function less emotionally and have weaker psychological resistance. These results are consistent with the research of other authors [41,42,43]. In patients with lung cancer, the symptoms imposing the greatest burden were appetite loss, affecting global quality of life (27%) and cognitive function (11%); fatigue, impacting role function (43%), emotional function (9%), and social function (27%); and dyspnea, which most strongly affected physical function (42%). Therefore, reducing these symptoms can improve the QoL and survival of lung cancer patients [44]. This study found a somewhat stronger and negative association between cough and work functioning in patients with lung cancer. These findings are in line with research demonstrating that cough primarily affects patients by causing negative emotions, impairing social relationships, and reducing quality of life [45]. Negative emotions include anger, worry, fear, depression, nervousness, anxiety, sensitivity and discomfort. There is a decrease in social contacts and a delay in returning to work, which can negatively affect the patient’s return to social life, which weakens their relationships with other people thus leading to poor social functioning. Research highlights a gap in knowledge regarding cough and underscores the importance of providing timely health education and professional medical care [45]. In lung cancer patients, symptoms such as cough, dyspnea, and chest pain can disrupt sleep, trigger anxiety or depression, and impair emotional and social functioning, reducing overall quality of life. Symptom burden and psychological distress interact in a vicious cycle, worsening both mood and functional ability. These findings emphasize the importance of effective symptom management and psychosocial support.

There are several limitations to this study. The study was conducted in a single center, which may reflect the specificities of the local population, care organization, and clinical practice, and it was conducted on a small number of patients. Also, the effects of comorbidities were not assessed, which we believe could influence the results of the study, and most patients had different therapeutic treatments at different stages of the disease. The study only classifies smoking status into “current smoker/former smoker/non-smoker” and does not collect key information such as smoking duration, daily smoking amount, and smoking cessation duration. These indicators may affect postoperative recovery. Therefore, the results cannot be generalized to the wider population or to other institutional settings without additional, multicenter studies with larger samples. The results of this study may serve to design the direction of interventions that will improve the QoL of patients with lung cancer.

## 5. Conclusions

The results of the values on the EORCT QLQ-C30 scale showed that patients with NSCLC assessed their health status better and evaluated the functioning scale better. In the evaluation of the EORCT QLQ-LC13 symptom scale, patients with SCLC assessed more pronounced cough, dyspnea, dysphagia, neuropathy and pain as the most frequent and strongest symptoms. QoL is becoming an increasingly important indicator for determining the short-term and long-term impact of both surgery and oncology in the treatment of patients with lung cancer. The results of this research indicate the need to pay special attention to symptom control and psychological support to patients in all phases of lung cancer treatment in clinical practice so that patients have better QoL.

## Figures and Tables

**Table 1:** Patient characteristics.

	N (%)
Gender	
Male	54 (84)
Female	10 (16)
Age	
31–45 years old	2 (2)
46–65 years old	31 (49)
66 and older	31 (49)
Place of residence	
City	41 (64)
Rural area	23 (36)
Education level	
Primary school	7 (11)
Secondary education	43 (67)
Higher vocational education	10 (16)
College education	4 (6)
Marital status	
Married or in a marriage-like relationship	43 (68)
Divorced or not living in a marriage-like relationship	8 (13)
Widow/er	9 (14)
Single/unmarried	3 (5)
Employed	28 (44)
Experienced pneumothorax	12 (19)
Family history of lung cancer	18 (28)
Smoking	
Active smoker	25 (39)
Used to smoke	24 (38)
Never smoked	15 (23)
Neoadjuvant therapy	29 (45)
Oncological therapy (*n* = 29)	
Chemotherapy	20 (69)
Chemotherapy and radiotherapy	9 (31)
Stage of lung cancer (*n* = 64)	
I	50 (78)
II	13 (20)
III	1 (2)
Type of cancer	
non-small cell lung cancer (NSCLC)	54 (84)
Small cell lung cancer (SCLC)	10 (16)

**Table 2:** QLQ-C30 scale values in relation to cancer type.

	Median (Interquartile Range) in Relation to Cancer Type		*p* *
	Non-Small Cell Lung Cancer (NSCLC) (*n* = 54)	Small Cell Lung Cancer (SCLC) (*n* = 10)	
General health status	50 (33.33–66.67)	33.33 (16.67–33.33)	<0.001
Physical functioning	73.33 (53.33–86.67)	53.33 (40–66.67)	0.004
Work functioning	50 (50–66.67)	50 (33.33–58.33)	0.13
Emotional functioning	41.67 (41.67–58.33)	33.33 (29.17–41.67)	0.005
Cognitive functioning	83.33 (66.67–100)	66.67 (66.67–91.67)	0.13
Social functioning	50 (33.33–66.67)	33.33 (16.67–50)	0.07
Fatigue	44.44 (33.33–58.33)	66.67 (50–66.67)	0.005
Nausea/vomiting	0 (0–16.67)	16.67 (0–33.33)	0.04
Pain	50 (33.33–50)	66.67 (50–66.67)	0.004
Breathing difficulties	33.33 (33.33–66.67)	66.67 (50–66.67)	0.03
Sleep disorders	66.67 (33.33–66.67)	66.67 (66.67–66.67)	0.11
Appetite loss	33.33 (0–33.33)	33.33 (33.33–66.67)	0.005
Constipation	33.33 (0–33.33)	33.33 (0–50)	0.51
Diarrhea	0 (0–33.33)	33.33 (0–33.33)	0.06
Financial difficulties	66.67 (66.67–100)	66.67 (66.67–100)	0.09
Functionality scale	61.11 (48.89–71.11)	48.89 (36.67–55.56)	0.01
Symptom scale	38.46 (30.77–46.15)	51.28 (41.03–58.97)	0.002

* Mann–Whitney U test.

**Table 3:** QLQ-C30 scale values in relation to pneumothorax.

	Median (Interquartile Range) in Relation to Pneumothorax		*p* *
	Experienced Pneumothorax (*n* = 12)	Did Not Experience Pneumothorax (*n* = 52)	
General health status	29.17 (10.42–62.5)	50 (33.33–66.67)	0.04
Physical functioning	80 (56.67–86.67)	66.67 (53.33–80)	0.21
Work functioning	41.67 (0–62.5)	50 (50–66.67)	0.03
Emotional functioning	41.67 (33.33–56.25)	41.67 (35.42–58.33)	0.44
Cognitive functioning	66.67 (50–100)	83.33 (66.67–100)	0.12
Social functioning	33.33 (4.17–62.5)	50 (33.33–66.67)	0.08
Fatigue	50 (36.11–75)	44.44 (44.44–66.67)	0.35
Nausea/vomiting	0 (0–16.67)	0 (0–33.33)	0.49
Pain	41.67 (33.33–79.17)	50 (33.33–66.67)	0.92
Breathing difficulties	66.67 (33.33–66.67)	50 (33.33–66.67)	0.16
Sleep disorders	66.67 (33.33–66.67)	66.67 (33.33–66.67)	0.55
Appetite loss	33.33 (0–33.33)	33.33 (33.33–33.33)	0.70
Constipation	0 (0–25)	33.33 (0–33.33)	0.02
Diarrhea	16.67 (0–33.33)	0 (0–33.33)	0.85
Financial difficulties	83.33 (41.67–100)	66.67 (66.67–91.67)	0.41
Functionality scale	53.33 (42.22–66.67)	57.78 (48.89–70.56)	0.24
Symptom scale	38.46 (26.92–58.33)	38.46 (33.33–48.08)	0.84

* Mann–Whitney U test.

**Table 4:** QLQ-C30 scale values in relation to smoking.

	Median (Interquartile Range) in Relation to Smoking			*p* *
	Active Smoker (*n* = 25)	Former Smoker (*n* = 24)	Non-Smoker (*n* = 15)	
General health status	33.33 (33.33–62.5)	33.33 (20.83–66.67)	66.67 (50–66.67)	0.04
Physical functioning	73.33 (53.33–86.67)	66.67 (53.33–80)	66.67 (53.33–80)	0.82
Work functioning	50 (33.33–66.67)	50 (33.33–66.67)	66.67 (50–83.33)	0.07
Emotional functioning	41.67 (41.67–50)	45.83 (33.33–66.67)	41.67 (33.33–58.33)	0.85
Cognitive functioning	83.33 (66.67–100)	66.67 (50–100)	100 (66.67–100)	0.26
Social functioning	50 (33.33–50)	50 (20.83–66.67)	50 (33.33–66.67)	0.78
Fatigue	44.44 (33.33–66.67)	44.44 (44.44–66.67)	44.44 (33.33–55.56)	0.49
Nausea/vomiting	16.67 (0–33.33)	0 (0–16.67)	0 (0–33.33)	0.56
Pain	50 (33.33–66.67)	50 (33.33–66.67)	50 (33.33–50)	0.29
Breathing difficulties	66.67 (33.33–66.67)	66.67 (33.33–66.67)	33.33 (33.33–66.67)	0.63
Sleep disorders	66.67 (33.33–66.67)	66.67 (33.33–66.67)	66.67 (33.33–66.67)	0.56
Appetite loss	33.33 (33.33–33.33)	33.33 (0–33.33)	33.33 (33.33–33.33)	0.50
Constipation	33.33 (0–33.33)	33.33 (0–33.33)	0 (0–33.33)	0.57
Diarrhea	33.33 (0–33.33)	0 (0–33.33)	0 (0–33.33)	0.34
Financial difficulties	66.67 (66.67–100)	66.67 (33.33–100)	66.67 (66.67–66.67)	0.73
Functionality scale	55.56 (46.67–68.89)	57.78 (42.22–71.11)	60 (48.89–71.11)	0.66
Symptom scale	43.59 (33.33–53.85)	35.9 (26.92–48.08)	38.46 (30.77–43.59)	0.21

* Kruskal–Wallis test.

**Table 5:** QLQ-LC13 scale values in relation to cancer type.

	Median (Interquartile Range) in Relation to Cancer Type		*p* *
	Non-Small Cell Lung Cancer (NSCLC) (*n* = 54)	Small Cell Lung Cancer (SCLC) (*n* = 10)	
Symptom scale			
Cough	33.33 (33.33–33.33)	33.33 (33.33–66.67)	0.02
Hemoptysis	0 (0–0)	0 (0–0)	0.60
Dyspnea	44.44 (22.22–55.56)	55.56 (44.44–61.12)	0.03
Oral pain	0 (0–33.33)	0 (0–33.33)	0.36
Dysphagia	0 (0–0)	0 (0–33.33)	0.005
Peripheral neuropathy	33.33 (0–33.33)	33.33 (16.67–66.67)	0.04
Alopecia	0 (0–0)	0 (0–33.33)	0.07
Chest pain	33.33 (33.33–33.33)	66.67 (33.33–66.67)	<0.001
Arms or shoulder pain	33.33 (0–33.33)	33.33 (33.33–66.67)	<0.001
Pain in other parts of the body	0 (0–33.33)	33.33 (0–50)	0.005
If pain medication was taken-pain reduction score	66.67 (41.67–66.67)	66.67 (41.67–91.67)	0.51

* Mann–Whitney U test.

**Table 6:** QLQ-LC13 scale values in relation to neoadjuvant therapy.

	Median (Interquartile Range) in Relation to Preoperative Oncological Therapy		*p* *
	Received Preoperative Oncological Therapy (*n* = 29)	Did Not Receive Preoperative Oncological Therapy (*n* = 35)	
Symptom scale			
Cough	33.33 (33.33–33.33)	33.33 (33.33–66.67)	0.07
Hemoptysis	0 (0–0)	0 (0–0)	0.94
Dyspnea	55.56 (22.22–55.56)	44.44 (30.55–55.56)	0.79
Oral pain	0 (0–33.33)	0 (0–0)	0.14
Dysphagia	0 (0–0)	0 (0–0)	0.94
Peripheral neuropathy	33.33 (0–33.33)	33.33 (0–33.33)	0.79
Alopecia	0 (0–33.33)	0 (0–0)	0.10
Chest pain	33.33 (33.33–66.67)	33.33 (33.33–41.67)	0.74
Arms or shoulder pain	33.33 (0–33.33)	33.33 (0–33.33)	0.65
Pain in other parts of the body	0 (0–33.33)	0 (0–33.33)	0.33
If pain medication was taken—pain reduction score	66.67 (33.33–66.67)	66.67 (66.67–91.67)	0.04

* Mann–Whitney U test.

**Table 7:** Correlation of symptoms with the QLQ-LC13 scale.

	Spearman’s Correlation Coefficient Rho (*p* Value)										
	Cough	Hemoptysis	Dyspnea	Oral Pain	Dysphagia	Peripheral Neuropathy	Alopecia	Chest Pain	Arms or Shoulder Pain	Pain in Other Parts of the Body	Pain Reduction Score
General health status	−0.446 (<0.001)	−0.378 (<0.001)	−0.443 (<0.001)	−0.116 (0.37)	−0.320 (0.01)	−0.263 (0.04)	−0.221 (0.08)	−0.405 (<0.001)	−0.471 (<0.001)	−0.380 (<0.001)	−0.379 (0.01)
Physical functioning	−0.237 (0.06)	−0.128 (0.32)	−0.367 (<0.001)	−0.174 (0.18)	−0.277 (0.03)	−0.403 (<0.001)	−0.238 (0.06)	−0.383 (<0.001)	−0.305 (0.02)	−0.521 (<0.001)	−0.066 (0.66)
Work functioning	−0.520 (<0.001)	−0.177 (0.17)	−0.206 (0.11)	−0.16 (0.21)	−0.229 (0.07)	−0.097 (0.45)	−0.363 (<0.001)	−0.248 (0.05)	−0.314 (0.01)	−0.238 (0.07)	−0.288 (0.05)
Emotional functioning	−0.151 (0.24)	−0.102 (0.43)	−0.485 (<0.001)	−0.065 (0.62)	−0.184 (0.15)	−0.417 (<0.001)	−0.038 (0.77)	−0.561 (<0.001)	−0.488 (<0.001)	−0.509 (<0.001)	−0.210 (0.15)
Cognitive functioning	−0.261 (0.04)	−0.283 (0.03)	−0.281 (0.03)	−0.176 (0.17)	−0.124 (0.34)	−0.244 (0.06)	−0.277 (0.03)	−0.315 (0.01)	−0.525 (<0.001)	−0.366 (<0.001)	−0.270 (0.06)
Social functioning	−0.367 (<0.001)	−0.048 (0.71)	−0.453 (<0.001)	−0.075 (0.56)	−0.193 (0.13)	−0.24 (0.06)	−0.155 (0.23)	−0.420 (<0.001)	−0.479 (<0.001)	−0.378 (<0.001)	−0.260 (0.07)
Fatigue	0.319 (0.01)	0.122 (0.35)	0.326 (0.01)	0.204 (0.12)	0.291 (0.02)	0.293 (0.02)	0.336 (0.01)	0.381 (<0.001)	0.407 (<0.001)	0.461 (<0.001)	0.020 (0.89)
Nausea/vomiting	0.074 (0.56)	0.112 (0.38)	0.162 (0.21)	0.321 (0.01)	0.347 (0.01)	0.282 (0.03)	0.406 (<0.001)	0.126 (0.33)	0.248 (0.05)	0.443 (<0.001)	0.052 (0.73)
Pain	0.287 (0.02)	0.159 (0.22)	0.479 (<0.001)	0.226 (0.08)	0.366 (<0.001)	0.426 (<0.001)	0.218 (0.09)	0.568 (<0.001)	0.429 (<0.001)	0.550 (<0.001)	0.122 (0.41)
Breathing difficulties	0.341 (0.01)	−0.077 (0.55)	0.339 (0.01)	0.07 (0.59)	0.137 (0.29)	0.287 (0.02)	0.176 (0.17)	0.377 (<0.001)	0.436 (<0.001)	0.359 (<0.001)	0.210 (0.15)
Sleep disorders	0.112 (0.38)	−0.048 (0.71)	0.448 (<0.001)	−0.053 (0.68)	0.151 (0.24)	0.205 (0.11)	0.139 (0.28)	0.286 (0.02)	0.232 (0.07)	0.298 (0.02)	0.076 (0.61)
Appetite loss	0.325 (0.01)	−0.023 (0.86)	0.426 (<0.001)	0.216 (0.09)	0.302 (0.02)	0.238 (0.06)	0.289 (0.02)	0.265 (0.04)	0.129 (0.32)	0.513 (<0.001)	−0.022 (0.88)
Constipation	0.145 (0.26)	0.158 (0.23)	0.200 (0.12)	−0.103 (0.43)	0.050 (0.70)	0.094 (0.47)	0.040 (0.76)	0.168 (0.20)	0.02 (0.88)	0.095 (0.47)	0.008 (0.96)
Diarrhea	−0.053 (0.68)	−0.047 (0.72)	0.089 (0.49)	0.357 (<0.001)	0.506 (<0.001)	0.254 (0.05)	0.385 (<0.001)	0.187 (0.15)	0.15 (0.24)	0.318 (0.01)	0.016 (0.91)
Financial difficulties	0.201 (0.11)	−0.103 (0.43)	0.367 (<0.001)	−0.045 (0.73)	0.326 (0.01)	0.119 (0.36)	0.164 (0.20)	0.267 (0.04)	0.165 (0.20)	0.339 (0.01)	0.022 (0.88)
Functionality scale	−0.393 (<0.001)	−0.193 (0.14)	−0.464 (<0.001)	−0.149 (0.25)	−0.254 (0.05)	−0.366 (<0.001)	−0.238 (0.06)	−0.479 (<0.001)	−0.524 (<0.001)	−0.530 (<0.001)	−0.251 (0.09)
Symptom scale	0.301 (0.02)	0.100 (0.44)	0.425 (<0.001)	0.243 (0.06)	0.412 (<0.001)	0.371 (<0.001)	0.413 (<0.001)	0.446 (<0.001)	0.440 (<0.001)	0.590 (<0.001)	0.134 (0.36)

## Data Availability

The data presented in this study are available on request from the corresponding author due to specifics of the patients.

## Abbreviations

The following abbreviations are used in this manuscript:

QoL	Quality of life
QLQ	Quality of life questionnaire
LDCT	Low-dose computed tomography
NSCLC	Non-small cell lung cancer
SCLC	Small cell lung cancer
EORTC	European Organization for Research and Treatment of Cancer
